# Calcitonin as an adjunct for phantom limb pain

**DOI:** 10.1371/journal.pone.0351739

**Published:** 2026-06-16

**Authors:** Sneha Vidyasagar, Kanakamani Jeyaraman, Syeda Farah Zahir, Paul Varghese

**Affiliations:** 1 Logan Endocrine and Diabetes Service, Logan, QLD, Australia; 2 Department of Diabetes and Endocrinology, Princess Alexandra Hospital, Brisbane, QLD, Australia; 3 QCIF Biostatistics, Brisbane, QLD, Australia; 4 Department of Geriatric Medicine, Princess Alexandra Hospital, Brisbane, QLD, Australia; 5 The University of Queensland School of Medicine, Brisbane, QLD, Australia; Kwara State University, NIGERIA

## Abstract

**Objective:**

This retrospective study evaluated calcitonin as an adjunct therapy for reducing phantom limb pain (PLP) following lower limb amputation.

**Method:**

The study included 35 patients who received at least 3 days of calcitonin treatment between January 1, 2017, and December 31, 2023. We collected demographic data and pain ratings (intensity, distress, and interference with activity) before and after calcitonin treatment. Descriptive statistics and paired t-tests analysed the data, with a two-way repeated measures ANOVA used to compare outcomes between patients with and without diabetes. Raw and Standardized mean differences (Cohen’s d) are presented for each measure.

**Results:**

The average age of participants was 57.09 years (SD = 13.66), with 40% female. Amputation types were below-knee (65.7%), above-knee (25.7%), and other (8.6%). The main causes of amputation included diabetic foot infection (25.7%), peripheral vascular disease (34.3%), trauma (25.7%), and other (14.3%). Ten participants had diabetes, and 20% had depression.

Calcitonin was associated with significant reductions in pain outcomes from pre- to post-intervention (Day 0 to Day 7), with calcitonin given from day 1–3. Mean pain intensity decreased from 6.41 to 5.24 (Cohen’s d = 0.66); p = 0.02), and pain-related distress decreased from 5.85 to 4.81 (Cohen’s d = 0.71; p = 0.014). Perceived pain relief scores increased from 33.69 to 58.21, indicating greater patient-reported pain relief following treatment (Cohen’s d = 0.53; p = 0.035). No significant differences in pain intensity or distress were observed between patients with and without diabetes. Additionally, there was no significant change in the Oral Morphine Equivalent Daily Dose (p = 0.94).

**Conclusion:**

In conclusion, calcitonin significantly reduced perceived pain intensity and pain-related distress scores while increasing perceived pain relief scores (i.e., patients’ reported degree of pain reduction), with similar effects observed in both patients with and without diabetes.

## Background

Phantom Limb pain (PLP) was first hypothesised in 1551 and is described as a painful sensation in a portion of the body that has been amputated [1]. Most patients describe this as a burning, stinging, aching or piercing pain. It is estimated that 65% of amputees will develop PLP within one month of amputation, 82% within one year, and 87% throughout their lifetime [1]. About 13–16% of patients with PLP experience chronic pain with the mean duration being 26 years with 50% experiencing one episode per week [2]. The average pain intensity 3 months postoperatively is reported as 50–79 on a Visual Analogue Scale (0–100) [2].

The exact aetiology of PLP remains unknown but is thought to be due to involvement of both the peripheral and central nervous system [1]. It is believed that when the peripheral nerves are amputated, the remaining axons attempt to innervate the “missing” limb which results in the formation of a neuroma [3]. In neuromas, nerve fibres have increased sodium channels, leading to hyperexcitability and heightened pain sensitivity. In the spinal cord, elevated NMDA activity in the dorsal horn makes it more reactive to pain-triggering substances like substance P and neurokinins. In the brain, cortical remapping causes neurons from adjacent areas to take over the region originally responsible for the amputated limb, resulting in pain sensations when these nearby areas are activated.

Although there are multiple proposed treatment options, none have been extensively studied specifically for PLP and the reported studies have conflicting results [1,2].

Calcitonin is a polypeptide hormone consisting of 32 amino acids [3]. Calcitonin receptors are broadly distributed not only in osteoclasts but also in brain, ovary, kidney, stomach, and skeletal muscles. It has been trialled as an adjunct analgesic in non-specific lumbar back pain, diabetes-associated neuropathic pain, headaches, postoperative pain and complex regional pain syndrome. The analgesic mechanisms of calcitonin remain poorly understood. In rats, calcitonin increases 5-HT_1A_ receptors, which is thought to be reduced in chronic pain. Furthermore, calcitonin inhibits abnormal expression of sodium channels in peripheral nerves, which provides an analgesic effect. Hence, calcitonin’s modulation of voltage-gated sodium channels may reduce peripheral nerve firing after amputation, while its serotonergic effects may enhance descending inhibitory control, addressing both peripheral and central drivers of phantom limb pain. These dual actions strengthen its biological plausibility as an adjunctive treatment for PLP [1–3].

A systematic review identified six published studies that reported the use of calcitonin for PLP [2]. Overall, there appears to be treatment benefit of calcitonin as an adjunct pain medication for PLP. Current recommendations include administration of Calcitonin intravenously as a 200 IU infusion, for 3–5days.

## Objectives

The aim of this study was to evaluate the efficacy of calcitonin as an adjunct therapy for PLP following lower limb amputation.

### Primary objective

To assess the effectiveness of calcitonin in reducing PLP, specifically:

a) Improvement in average pain intensity before and after treatment.
b) Improvement in distress levels before and after treatment.
c) The degree of pain relief achieved by treatment.

### Secondary objectives

To explore differences between patients with and without diabetes:

a) Changes in pain intensity scores pre- and post-calcitonin.
b) Changes in distress scores pre- and post-calcitonin.

To assess changes in the Oral Morphine Equivalent Daily Dose (OMEDD) before and after calcitonin treatment.

## Methods

This retrospective study aimed to investigate the efficacy of calcitonin as a pain adjunct in the management of phantom limb pain (PLP) in patients who developed PLP following amputation. We reviewed the electronic medical records of all patients who underwent amputation and received calcitonin at the Princess Alexandra Hospital between 1 January 2017 and 31 December 2023. Demographic information, including age, gender, ethnicity and comorbidities was recorded and all information was de-identified at the time of data collection. Information related to the cause of amputation, details of analgesia received for management of pain including calcitonin therapy, oral, intravenous, epidural or nerve blocks was also collected. Data was collected between 1 April 2024–1 August 2024.

The study design involved comparing the pre calcitonin and post calcitonin pain ratings (intensity, distress and perceived pain relief) in patients who received calcitonin as an adjunct therapy post amputation. All adult patients who received calcitonin therapy for the management of PLP were eligible for inclusion. Calcitonin was administered intravenously over 3 days, using either 200 IU daily for 3 days or 100 IU on day 1 followed by 200 IU daily thereafter. This depended on prior exposure and tolerability. Patients who did not have pain scores documented were excluded (8 participants).

The primary outcome was documented as pain rating scores based on the British Pain Society Pain Rating Scale at Day 0 (pre calcitonin) and Day 7. This included questions on ‘how intense is your pain now’, ‘how intense was your pain on average last week’, ‘how distressing is your pain now’,’ how distressing was your pain on average last week’ and ‘ how much treatment took away the pain’. There is currently no single universally accepted, gold-standard, and fully standardized tool for phantom limb pain (PLP).The Oral Morphine Equivalent Daily Dose was calculated pre and post calcitonin infusion based on the documented medication records (i.e., concurrent analgesics given during the same period) using the opioid dose equivalence table published by the Australian and New Zealand college of Anaesthetists [4].

Ethics approval was granted for the study (HREC/2024/QMS/105190), and the requirement for informed consent was waived.

## Statistical methods

Descriptive statistics summarized the population, with continuous variables analyzed using measures of central tendency and spread and categorical variables were described using counts and proportions. Normality was assessed using the Shapiro-Wilk’s test. Both raw mean differences (with confidence intervals) and standardized effect sizes were calculated for all comparisons to provide a comprehensive understanding of the findings. Differences in normally distributed data (pain intensity and distress) were analyzed using paired t-tests, while non-normal data (pain relief scores) were assessed with the Wilcoxon Signed-Rank test. Normality of difference scores was examined using the Shapiro-Wilk’s test. Homogeneity of variances was assessed using Levene’s test.

For secondary outcomes, changes across time by diabetes status were assessed using a two-way repeated measures ANOVA (time X diabetes). Assumptions of sphericity were assessed (where applicable), and homogeneity of variances was tested using Levene’s test.

Effect sizes for parametric tests were calculated as Cohen’s d with 95% CIs (effsize package, paired = TRUE), and for non-parametric tests as r = Z/√N.

Results are presented as mean differences (or median differences for non-parametric tests) with corresponding confidence intervals, test statistics, exact p-values, and effect sizes. Cohen’s d values were interpreted as small (0.2), medium (0.5), or large (0.8), while r values were interpreted as small (0.1), medium (0.3), or large (0.5) according to Cohen’s conventions.

### Statistical analysis

Descriptive statistics summarized the population, with continuous variables presented using measures of central tendency and spread (mean ± SD for normal data; median [IQR] for non-normal data), and categorical variables described using counts and proportions. Normality was assessed using the Shapiro-Wilk test. Both raw mean differences (with confidence intervals) and standardized effect sizes were calculated for all comparisons to provide a comprehensive understanding of the findings. The threshold for statistical significance was set at p < 0.05.

Differences in normally distributed data (pain intensity and distress) were analyzed using paired t-tests. For non-normal data (pain relief scores), the Wilcoxon signed-rank test was employed. Normality of difference scores was examined using the Shapiro-Wilk test and visual inspection of Q-Q plots.

Effect sizes for parametric tests were calculated as Cohen’s d with 95% confidence intervals. For non-parametric tests, effect sizes were calculated as r = Z/ √N, where Z is the standardized test statistic from the Wilcoxon test and N is the number of observations.

To account for multiple testing across several outcomes, we designated (pain intensity and distress) as the primary outcomes, with all others considered secondary/exploratory. Greater emphasis was placed on effect sizes and confidence intervals rather than solely on p-values. No adjustment was made for multiple comparisons, as these analyses were exploratory. All p-values are presented unadjusted to avoid inflating Type II error, and findings should be interpreted in the context of effect sizes and confidence intervals to facilitate transparent interpretation. All statistical analyses were performed in R (Version 4.3.2).

## Results

Between 1 January 2017 and 31 December 2023, 35 patients were enrolled in the study and had a mean age of 57.09 years (SD = 13.66), 40% of whom were female. Most amputations were below-knee (65.7%), followed by above-knee (25.7%), and others (8.6%). Ten participants had diabetes. The main causes of amputation were diabetic foot infection (25.7%), peripheral vascular disease (34.3%), trauma (25.7%), and other (14.3%). Additionally, 20% had depression and 8.6% had opiate dependence (Table 1). Primary outcomes looked at the average pain intensity and distress levels at day 0 (prior to the intervention) and day 7 (with calcitonin administered between day 1–3) as well as how much treatment relieved the pain comparing day 0 to day 7, which was expressed as a percentage from 0% to 100%.

**Table 1:** Summary of demographics of study population.

	Overall (N = 35)
**Age**	
Mean (SD)	57.09 (13.66)
Median (Q1, Q3)	58.00 (45.00, 65.50)
Min – Max	28.00 - 88.00
Missing	0.00
**Sex**	
Female	14.00 (40.0%)
Male	21.00 (60.0%)
Missing	0.00
**SEIFA**	
Mean (SD)	4.14 (2.88)
Median (Q1, Q3)	3.00 (2.00, 6.50)
Min – Max	1.00 - 10.00
Missing	0.00
**Duration of Diabetes**	
Mean (SD)	3.86 (7.00)
Median (Q1, Q3)	0.00 (0.00, 5.50)
Min – Max	0.00 - 23.00
Missing	0.00
**Dyslipidaemia**	
No	23.00 (65.7%)
Unsure	1.00 (2.9%)
Yes	11.00 (31.4%)
Missing	0.00
**Ischaemic Heart Disease**	
No	27.00 (77.1%)
Unsure	1.00 (2.9%)
Yes	7.00 (20.0%)
Missing	0.00
**Hypertension**	
No	25.00 (71.4%)
Unsure	1.00 (2.9%)
Yes	9.00 (25.7%)
Missing	0.00
**Previous Stroke**	
No	32.00 (91.4%)
Unsure	1.00 (2.9%)
Yes	2.00 (5.7%)
Missing	0.00
**Depression**	
No	27.00 (77.1%)
Unsure	1.00 (2.9%)
Yes	7.00 (20.0%)
Missing	0.00
**Opiate Dependance**	
No	31.00 (88.6%)
Unsure	1.00 (2.9%)
Yes	3.00 (8.6%)
Missing	0.00
**Level of amputation**	
Below Knee Amputation	23.00 (65.7%)
Above Knee Amputation	9.00 (25.7%)
Other	3.00 (8.6%)
Missing	0.00
**Reason for Amputation**	
Peripheral Vascular Disease	12.00 (34.3%)
Diabetic Foot infection	9.00 (25.7%)
Trauma	9.00 (25.7%)
Infection	2.00 (5.7%)
Thrombus	3.00 (8.6%)
**Previous amputation**	
Yes	2.00 (5.7%)
No	33.00 (94.3%)
Missing	0.00
**Pre Calcitonin OMED**	
Median (Q1, Q3)	90.00 (26.50, 112.50)

**Fig 1:**
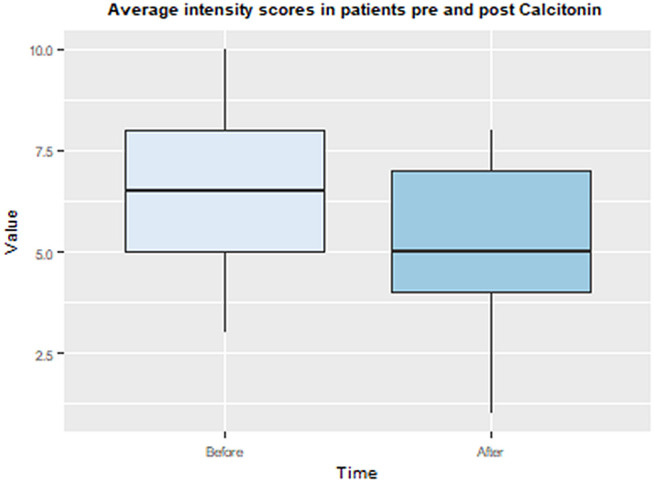
Improvement in average pain intensity scores pre and post calcitonin.

There was a statistically significant reduction in perceived pain intensity from pre-intervention (M = 6.41, SD = 1.75) to post-intervention (M = 5.24, SD = 1.77, p = 0.02), with a mean difference of 1.19 (95% CI = 0.21 to 2.16), t(23) = 2.52, p = 0.02. The effect size was medium (Cohen’s d = 0.66, 95% CI = 0.08 to 1.25), indicating a moderate clinical effect (Table 2 and Fig 1).

**Table 2:** Summary table of outcomes showing pre and post scores, raw and standardized mean differences mean difference along with p-values.

Outcome	Before Mean (SD)	After Mean (SD)	Raw mean Diff (95% CI)	SMD (95% CI)	p-value
**Pain intensity**	6.41 (1.75)	5.24 (1.77)	1.19 (0.21 to 2.16)	0.66,(0.08 to 1.25)	0.02
**Pain Distress**	5.85 (1.74)	4.81 (1.96)	1.03 (0.29 to 2.32)	0.71 (0.11 to 1.30)	0.01
**Pain Relief**	30 (0-60)*	60 (47.5-76.25)*	30 (0-35)*	–	−0.04*

*Median scores, IQR were used for Pain relief.

*No adjustment was made for multiple comparisons. Findings should be interpreted with emphasis on effect sizes and confidence intervals. All three outcomes were pre-specified as primary based on distinct clinical domains.*

**Fig 2:**
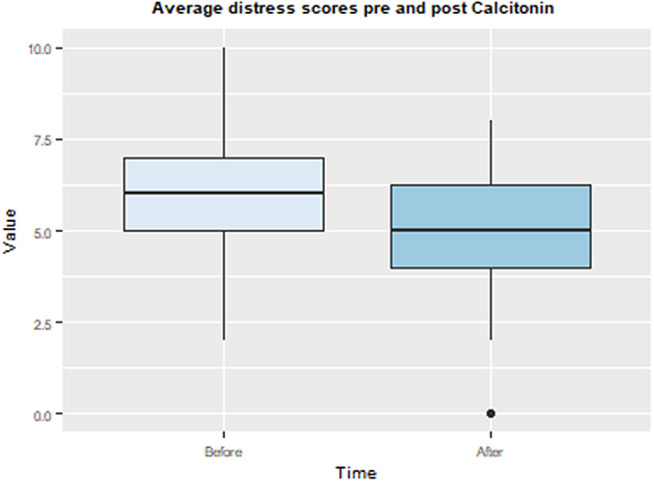
Improvement in average pain distress scores pre and post calcitonin.

Similarly, there was a statistically significant reduction in perceived distress caused by pain from pre-intervention (M = 5.85, SD = 1.74) to post-intervention (M = 4.81, SD = 1.96, p = 0.014) with a mean decrease of 1.03 (95% CI = 0.29 to 2.32), t(22) = 2.67, p = 0.01. The effect size was medium to large (Cohen’s d = 0.71, 95% CI = 0.11 to 1.30), indicating a moderate clinical effect (Table 2 and Fig 2).

**Fig 3:**
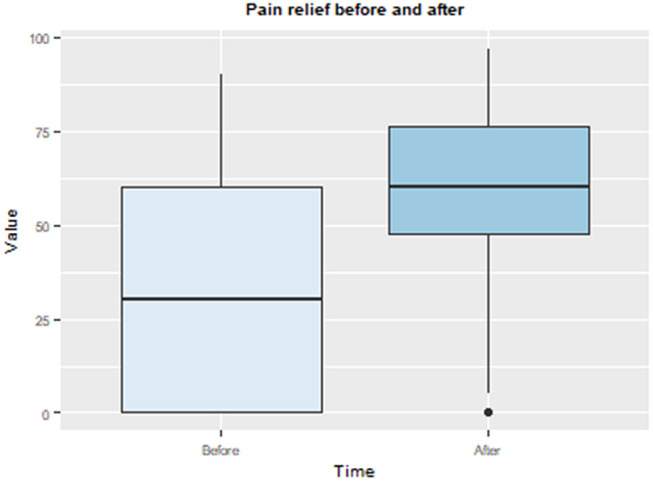
How much treatment took away the pain.

There was a statistically significant increase in how much treatment relieved (took away) the pain from pre-intervention (median = 30, IQR = 0–60) to post-intervention (median = 60, IQR = 47.50 to 76.25, p = 0.035) with a median increase of 30 units (95% CI: 0–35, V = 22.5, p-value = 0.04), representing a medium effect (r = 0.46) (Table 2 and Fig 3).

There was no difference in intensity score (F (1, 22.0) = 2.33, p = 0.141), and distress score (F (1, 21.0) = 2.67, p = 0.117) in patients with and without diabetes across time.

**Fig 4:**
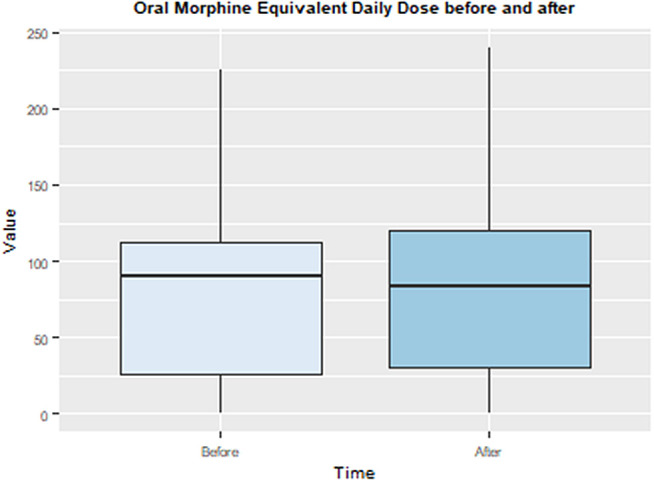
Oral morphine equivalent daily dose pre and post calcitonin.

The Oral Morphine Equivalent Daily Dose (OMEDD) showed no significant change (p = 0.94) pre calcitonin (median = 90), IQR (26.50 to 112.50) and post calcitonin (median = 83, IQR (30–120) (Table 3 and Fig 4).

**Table 3:** Summary statistics of oral morphine equivalent daily dose pre and post calcitonin.

Value	Before (N = 35)	After (N = 35)	Total (N = 70)
Median (Q1, Q3)	90.00 (26.50, 112.50	83.00 (30.00, 120.00)	86.50 (30.00, 120.00)
Min – Max	0.00 - 225.00	0.00 - 240.00	0.00 - 240.00
Missing	4.00	8.00	12.00

The median difference was 0 (bootstrap 95% CI (0–0), indicating no change for the typical participant. However, examination of individual responses revealed that 53.8% (14/26) showed no change, 23.1% (6/26) improved, and 23.1% (6/26) worsened. The overall Wilcoxon test was not significant (V = 44, p = 0.94), with a small effect size (r = 0.01).

## Discussion

This retrospective study examining the efficacy of calcitonin as an adjunctive treatment for phantom limb pain (PLP) in post-amputation patients showed statistically significant reductions in pain intensity, distress, and the perception of pain relief, suggesting a potential benefit of calcitonin therapy in addressing PLP. This is in keeping with a recent systematic review by Neumüller et al. which concluded that although relatively few studies report on the effectiveness of calcitonin for PLP, together they suggest an overall treatment benefit as an adjunctive therapy [2].

In our study we showed a statistically significant decrease in perceived pain intensity and distress. Importantly, the improvement in how much treatment was perceived to alleviate pain suggests that patients found the calcitonin therapy to have a beneficial impact on their pain management.

To the best of our knowledge there are no studies comparting the efficacy of calcitonin for PLP in those with and without diabetes. No statistically significant differences were observed between diabetic and non-diabetic patients over time. This lack of difference could be reflective of the fact that both groups may share common pathophysiological mechanisms related to the development of PLP post-amputation, however it is important to note that this was a small sample size with 10 participants with diabetes and 25 without. Studies have shown that the characteristics and intensity of PLP post-amputation remained similar in those with and without diabetes, irrespective of duration of diabetes and time since amputation [5].

A notable finding is the lack of change in OMEDD after calcitonin administration. This suggests that calcitonin, while effective in reducing pain intensity and distress, did not lead to a reduction in opioid doses. There could be several reasons for this. Firstly, opioids are effective for nociceptive pain, however their analgesic efficacy in neuropathic pain is subject to considerable uncertainty, which may explain the absence of a change in OMEDD [6]. Moreover, the presence of other types of post-operative pain—such as stump pain—during the immediate post-operative period may have attenuated the observed effects on narcotic consumption [7]. The interaction between pain sources may have masked the impact of the intervention on narcotic consumption, and this limitation should be considered when interpreting the findings. Secondly, given this was a retrospective study the reduction in opiate dose was heavily dependent on clinical judgement and there was no set protocol to assess and titrate analgesia and this could be explored further in future research. To the best of our knowledge there are no other studies suggesting that calcitonin can reduce other analgesic requirements for PLP, however in other conditions such as vertebral fractures, calcitonin was associated with statistically significantly less frequent analgesic use compared with placebo by week 1 and week 4 [8].

Strengths of our study include the prospective collection of data using a consistent questionnaire pre and post calcitonin administration, which minimises potential biases to enhance the reliability of the findings. Additionally, the documentation and calculation of opiate use was via documentation on electronic medical records, providing accurate and comprehensive data. Limitations include the fact that this is a small study with only 35 participants and no long-term follow-up of pain scores (i.e., 3–6 months post). Given this was retrospective the timing of calcitonin was not always immediately following amputation and in some cases several months after. This is reflective of the natural trajectory of PLP, given it does not always develop immediately post operatively and while 65% of amputees will develop PLP within one month of amputation some can develop it several months to years later. While some patients did not receive calcitonin immediately post operatively in our study, all but 2 patients received calcitonin within 12 months of amputation. We could consider future studies with a more regimented protocol to assess the benefit of calcitonin if given immediately post operatively rather than waiting for PLP to develop. In our hospital, the timing of the infusion depended on patient symptoms and lack of response to other treatment modalities. Previous studies have suggested that timing of calcitonin administration relative to symptom onset appears to play an important role in its analgesic properties for patients with PLP. For example, two randomized controlled trials showed that calcitonin has an early and often sustained analgesic benefit when administered either shortly after the initial onset of PLP or prior to the amputation. In contrast, studies describing the treatment of PLP with calcitonin at later timepoints produced heterogeneous results [2].

Patients without prior exposure were given a day 1 dose of 100U to minimise the risk of side effects which may cause them to abandon future treatment, followed by 200U on day 2 and 3. If they have had prior exposure then they were given 200U on days 1–3. The variation in dosage is around assessing tolerability, and future studies could look at stratifying this data depending on the dose and previous exposure.

It is important to also recognise, potential confounding from concurrent analgesic interventions and the natural postoperative pain trajectory that may have influenced the observed outcomes, and hence causality should therefore be interpreted cautiously.

The heterogeneity of the patient population, including varying levels of amputation and comorbidities, introduces another potential source of variability that could influence outcomes, particularly in terms of pain perception and narcotic use. Because this study was retrospective and lacked a control group, the observed improvements in pain scores cannot be confidently attributed to calcitonin alone. Furthermore, the modest overall sample size, particularly the very small diabetic subgroup (n = 10), limits statistical power, and therefore should be interpreted with caution. Due to the small sample size, we are unable to reliably provide further sub-analysis looking at how variables such as psychological comorbidities or opiate dependence may affect pain perception.

This study examined three primary outcomes without adjustment for multiple comparisons, which increases the risk of Type I error. However, we made this decision deliberately due to the small sample size and preliminary nature of the research, prioritizing detection of potentially meaningful effects over strict Type I error control. Importantly, we have emphasized effect sizes and confidence intervals throughout, providing a more complete picture than p-values alone.

This study highlights many gaps in the literature and gives rise to opportunities for future studies, ideally prospective and randomised, to further explore calcitonin’s role in PLP management and its interaction with other treatment modalities, especially in relation to opioid reduction. Additionally, alternative routes of calcitonin administration—such as intravenous (IV), intramuscular (IM), epidural, and intranasal, could be explored, as well as the optimal timing of calcitonin administration.

## Conclusion

This study suggests a potential benefit of calcitonin as an adjunct in the management of PLP. While it demonstrates significant improvements in perceived pain intensity, distress, and the subjective experience of pain relief, it did not show a corresponding decrease in opioid consumption (OMEDD). The absence of a significant difference between patients with and without diabetes suggests that calcitonin’s effects may transcend this variable. However, there is a need for prospective randomised studies to further establish the efficacy and optimal use of calcitonin in post amputation pain management and account for potential confounders inherent to this retrospective study.
